# A Distinct Fatty Acid Profile Underlies the Reduced Inflammatory State of Metabolically Healthy Obese Individuals

**DOI:** 10.1371/journal.pone.0088539

**Published:** 2014-02-10

**Authors:** Maude Perreault, Michael A. Zulyniak, Flavia Badoud, Susan Stephenson, Alaa Badawi, Andrea Buchholz, David M. Mutch

**Affiliations:** 1 Department of Human Health and Nutritional Sciences, University of Guelph, Guelph, Ontario, Canada; 2 Guelph Family Health Team, Yarmouth Medical Group, Guelph, Ontario, Canada; 3 Office of Biotechnology, Genomics and Population Health, Public Health Agency of Canada, Toronto, Ontario, Canada; 4 Department of Family Relations and Applied Nutrition, University of Guelph, Guelph, Ontario, Canada; GDC, Germany

## Abstract

**Background:**

Obesity is associated with numerous health complications; however, a subgroup of obese individuals (termed the metabolically healthy obese or MHO) appear to have lower risk for complications such as type 2 diabetes and cardiovascular disease. Emerging evidence suggests that MHO individuals have reduced inflammation compared to their metabolically unhealthy obese (MUO) counterparts. As it is recognized that fatty acids (FAs) have a strong relationship with inflammation, the current study aimed to uncover if the reduced inflammation observed in MHO individuals is mirrored by a more favourable FA profile.

**Methods:**

Fasted serum samples were collected from lean healthy (LH), MHO, and MUO participants (n = 10/group) recruited from the Diabetes Risk Assessment study. A panel of pro- and anti-inflammatory markers were measured by immunoassay. Total serum FA profiling, as well as the FA composition of circulating phospholipids (PL) and triglycerides (TG), was measured by gas chromatography. ANOVA and Mann-Whitney-Wilcoxon tests were used to assess statistical significance between the groups (P<0.05).

**Results:**

MHO and MUO individuals had similar BMI and body fat %; however, lipid parameters in MHO individuals more closely resembled that of LH individuals. MHO individuals had circulating levels of high sensitivity C-reactive protein (hsCRP) and interleukin-6 (IL-6) similar to LH individuals, while levels of platelet derived growth factor-ββ (PDGF-ββ) were intermediate to that of LH and MUO individuals. FA profiling analysis combined with discriminant analysis modelling highlighted a panel of nine FAs (consisting of three saturated, three monounsaturated, and three polyunsaturated FAs) in PL and TG fractions that distinguished the three groups. Specifically, saturated FA (myristic and stearic acids) levels in MHO individuals resembled that of LH individuals.

**Conclusion:**

Our results suggest that the reduced inflammatory state of MHO individuals compared to MUO individuals may stem, in part, from a more favourable underlying FA profile.

## Introduction

The low-grade chronic inflammation characteristic of obesity plays a significant role in the development of downstream complications, such as type 2 diabetes and cardiovascular disease [1]–[3]. However, evidence suggests that not all obese individuals are at a similar risk for these complications [4], [5]. Obese individuals who are seemingly protected from downstream complications are classified as metabolically healthy obese (MHO). While our molecular understanding of the MHO phenotype remains limited, clinical research has shown these individuals are more insulin sensitive and present a favourable lipid status compared to their metabolically unhealthy obese (MUO) counterparts (also referred to as “metabolically abnormal obese”) [6]. Recent observations also suggest that MHO individuals may have a reduced inflammatory status compared to MUO individuals [5], [7], [8].

Few studies have investigated the inflammatory profile associated with MHO. In 2005, Karelis *et al*. first noted that post-menopausal MHO women had lower levels of circulating high sensitivity C-reactive protein (hsCRP) and inflammation-sensitive protein alpha-1 antitrypsin (A1AT) compared to MUO women [7]. Subsequently, Klöting *et al.* reported that MHO individuals had lower circulating levels of various inflammatory markers (e.g., CRP, progranulin, chemerin, and retinol-binding protein-4) compared to MUO subjects [5]. More recently, Phillips and Perry demonstrated that MHO individuals had lower concentrations of a number of pro-inflammatory markers (e.g., complement component 3, CRP, tumour necrosis factor-α, interleukin-6, and plasminogen activator inhibitor-1) and higher adiponectin compared to MUO individuals of similar adiposity [8]. Together, these studies provide evidence that MHO have reduced inflammation compared to their MUO counterparts; however, the mechanisms responsible for this disparity remain to be elucidated.

It is now widely appreciated that FAs can influence whole-body inflammation by regulating the production and secretion of cytokines, chemokines, and eicosanoids [9], [10]; however, not all FAs act similarly. Saturated and *trans* fats tend to be positively associated with inflammation, while monounsaturated and polyunsaturated fats typically have beneficial effects [11]–[13]. As such, elucidating the FA profile in MHO individuals will provide important insight to help us better understand the basis for their reduced inflammatory state.

Total circulating levels of free FAs (i.e., FFAs) were reported to be lower in MHO compared to MUO individuals [14], [15]; however, it remains unknown if individual FA levels differ between MHO and MUO individuals. This is relevant given that past research has shown that measuring FAs in specific lipid fractions (e.g., phospholipid, PL; triglyceride, TG) can provide novel insight to help understand the changes in FA metabolism that are associated with inflammation [16], [17]. For example, Pietiläinen *et al.* employed a global FA profiling approach to show that expanding adipose tissue is characterized by a FA profile that may favour inflammation [17]. While such an approach has not been used to study MHO, this is warranted given that the expression of lipogenic genes was recently shown to differ between MHO and MUO individuals [15]. As such, we expect that using a FA profiling approach will generate novel insight to help understand if FAs contribute to the reduced inflammatory state seen in MHO individuals.

In the current study we first set out to confirm that MHO individuals from our cohort were characterized by a reduced inflammatory state and then subsequently examined whether this was associated with a distinct circulating FA profile. Together, the knowledge generated by this research will help unravel the underlying basis for the reduced level of inflammation seen in MHO individuals, and may ultimately be used to develop tailored dietary strategies to more appropriately manage obesity-related complications.

## Materials and Methods

### Study Population

Individuals were recruited into the Diabetes Risk Assessment (DRA) study (Clinical Trial N^o^ NCT01884714) from Guelph, Ontario and the surrounding communities using study posters and newspaper advertisements. Persons expressing interest in the study were screened over the phone and excluded if they met any one of the following criteria: 1) below 35 or above 70 years of age; 2) diagnosed with an acute or chronic autoimmune inflammatory disease, infectious disease, viral infection, and/or cancer; or 3) regular alcohol consumption exceeding 2 drinks/day (1 drink = 10 g alcohol). The research protocol was approved by the University of Guelph Research Ethics Board (REB#10AP033). All participants signed a written consent form.

### Anthropometric Measurements

All measures related to adiposity (i.e., height (m), body weight (kg), waist and hip circumferences (cm), fat mass (% and kg) and fat-free mass (% and kg)) were obtained in the Body Composition and Metabolism Laboratory at the University of Guelph (www.uoguelph.ca/bodycomp). Body mass (to the nearest 0.1 kg) was measured with subjects wearing only a bathing suit and swimming cap, using the digital BOD POD scale (BOD POD Air Displacement Body Composition system; Life Measurement Inc., CA, USA). The scale was calibrated weekly against standardized 20-lb weights. Height was measured to the nearest 0.5 cm, using a wall-mounted stadiometer (Seca Corp., Ontario, Canada). Body mass index (BMI) was calculated from height and mass (kg/m^2^).

Fat mass and fat-free mass were measured using the BOD POD. The instrument was calibrated twice in the morning of each data collection day: once with the test chamber empty and once by placing a cylinder of known volume (49.980 L) in the chamber. Raw body volume was measured with subjects wearing only a bathing suit and swimming cap, with no jewelry. Subjects were instructed to sit quietly, limit movement, and breathe normally while in the test chamber. Body volume was measured twice and the average was used to determine body density. If the 2 measurements differed by more than 150 mL, a third measurement was taken and the average of the 2 closest was then used. The final step involved in determining body density was the measurement of thoracic gas volume; subjects were instructed to sit quietly and plug their noses while breathing through a disposable tube connected to the rear of the instrument. The subjects were instructed to make 3 quick, light pants, after 4 or 5 normal breaths. Percentage body fat was calculated from density using the Siri equation. All measurements were performed by the same trained person. The coefficient of variation for percentage body fat measurements was 2.2±2.3%.

### Bioclinical Measurements

Blood samples were collected from all participants following an overnight fast (∼12 hrs). Serum samples were sent to LifeLabs Medical Laboratory Services (Guelph, ON, Canada) for the analysis of glucose (mmol/L), insulin (pmol/L), total-triglycerides (TG; mmol/L), total-cholesterol (Total-c; mmol/L), high-density lipoprotein cholesterol (HDL-c; mmol/L), low-density lipoprotein cholesterol (LDL-c; mmol/L), glycosylated haemoglobin (HbA1c), and hsCRP (mg/L). Estimates of the insulin sensitivity (HOMA-IR) and β-cell function (HOMA%B) were calculated using the HOMA Calculator v2.2.2 [18].

Systolic and diastolic blood pressure was measured (in duplicate) at rest using an automated blood pressure monitor (Intellisense, OMRON Healthcare, Bannockburn, Il, USA).

### Classification of Groups

Thirty participants were classified into LH, MHO, and MUO groups based on their adiposity and metabolic status (n = 10/group). Adiposity status was determined using the revised BMI cut-offs proposed by Shah and Braverman, where lean was considered <28 kg/m^2^ for males and <24 kg/m^2^ for females, and obese was considered ≥28 kg/m^2^ for males and ≥24 kg/m^2^ for females [19]. Metabolic status was determined using criteria adapted from that originally proposed by Karelis *et al.* [20] in order to account for sex-specific differences and medication. An individual was considered “metabolically healthy” if 3 or more of the following criteria were met: HDL-c >1.0 mmol/L for males and >1.3 mmol/L for females; TG <1.7 mmol/L without use of lipid-lowering drugs; Total-c <5.2 mmol/L; LDL-c <2.6 mmol/L; and HOMA-IR <1.95 without use of anti-diabetic drugs. Each group was comprised of 7 women and 3 men. LH, MHO, and MUO groups were matched for age, while the MHO and MUO groups were matched for BMI and percentage body fat.

### Inflammatory Marker Analysis

A panel of pro- and anti-inflammatory markers were measured in fasted serum samples. Interleukin-10 (IL-10), monocyte chemotactic protein-1 (MCP-1/CCL2), tumour necrosis factor-α (TNF-α), and high-molecular weight adiponectin (HMW adiponectin) were measured, in duplicate, using immunoassay kits according to the manufacturer’s instructions (BioLegend, San Diego, CA, USA or R&D Systems, Minneapolis, MN, USA) and read using a SynergyMX plate reader (Biotek, Winooski, VT, USA). Interleukin-6 (IL-6), interleukin-1 receptor antagonist (IL-1Ra), interferon-γ (IFN-γ), regulated upon activation normal T-cell expressed and secreted (RANTES/CCL5), platelet derived growth factor-ββ (PDGF-ββ), and interferon-γ inducible protein 10 (IP-10) were measured, in duplicate, by multiplex bead immunoassay and read using the Bio-Plex suspension array system according to the manufacturer’s recommendations (Bio-Rad, Mississauga, ON, Canada).

### Fatty Acid Analysis

All solvents and reagents were obtained from Fisher Scientific (Toronto, ON, Canada). The isolation, extraction, and quantification of total FAs, as well as fractionated FAs (i.e., in PL and TG fractions), from fasted serum samples were performed as previously described [21]. Briefly, samples were spiked with 10 µl of a 1 µg/µL C17∶0 internal standard. Both total and fractionated FAs were extracted with chloroform:methanol (2∶1, v/v). Samples were then flushed with nitrogen and placed at ∼4°C over night. The next day, samples used for total FA analysis were methylated at 100°C for 1.5 hrs. For the analysis of FAs in isolated lipid fractions, samples were spotted onto Silica-G TLC plates (Analtech, Newark, N.J., USA) and incubated for ∼45 min with petroleum ether, ethyl ether and acetic acid (80∶20∶1, v/v/v). PL and TG lipid bands were collected into separate tubes and methylated at 100°C for 1.5 hrs. All samples were analyzed using an Agilent Technologies 7890A GC system (Agilent Technologies, Mississauga, ON, Canada) with flame ionization detector. Peaks were identified by comparison to a panel of 49 FA methyl ester standards suspended in hexane (ranging from C8∶0 to C24∶1n9). Relative FA values were calculated as a % of total peak area. Absolute FA values were calculated by comparison of individual FA peaks to the internal standard C17∶0. As such, individual FA values (for both total and fractionated analyses) are reported as relative percentage (% FA ± SEM) and/or absolute (µg/100 µL of serum) values.

### Statistical Analysis

Anthropometric, bioclinical, and inflammatory parameters were analyzed with Prism 5 software (GraphPad, La Jolla, CA, USA). First, a non-parametric ANOVA Kruskal-Wallis test was used to measure significance between the three groups (P<0.05). Second, a post-hoc non-parametric Mann-Whitney-Wilcoxon test was used for pairwise group comparisons in cases when the initial ANOVA was statistically significant.

An Orthogonal Projections to Latent Structures-Discriminant Analysis (OPLS-DA) was used to distinguish the three phenotypes based on their FA profiles (mean centred and scaled using Pareto variance) (SIMCA v13.0.3.0, Umetrics AB, Umeå, Sweden). Three principal components were generated and cross-validated 7 times, where 1/7^th^ of the data was randomly left out for each round of validation. Analysis of variance of cross-validated residuals (CV-ANOVA) was performed for each OPLS-DA in order to assess the reliability of the predictive model. FAs with the greatest variability between the three groups were identified using Variables of Importance in Projection (VIP) >1 [22]. FAs meeting our VIP cut-off were then individually assessed between the three groups using a non-parametric ANOVA Kruskal-Wallis test (P<0.05). When significance was observed for a FA, a post-hoc Mann-Whitney-Wilcoxon test was subsequently used for pairwise group comparisons (JMP Genomics v5.1, SAS Institute, Cary, NC, USA). FAs meeting our VIP cut-off were also examined by linear regression (adjusted for sex) with measures of adiposity (BMI and body fat %) using JMP Genomics software.

## Results

### Characteristics of the Groups

General characteristics of the three groups are outlined in **Table 1**. Briefly, the three groups (LH, MHO, and MUO) were matched for age, while the obese groups were matched for BMI, waist-to-hip ratio, and body fat (both % and kg). The MHO and LH groups had significantly lower circulating levels of Total-c and LDL-c compared to the MUO group. In contrast, no differences were seen between the MHO and MUO groups for HDL-c and TG levels. Consequently, the MHO group had a Total-c/HDL-c ratio intermediate to that of LH and MUO groups. The MHO and MUO groups were not different for fasted glucose levels or HbA1c. However, the MHO group had fasted insulin and HOMA-IR values intermediate to that of LH and MUO (P = 0.09 for both parameters), suggesting a trend for higher insulin sensitivity in MHO individuals.

**Table 1:** Study population characteristics.

Parameters	LH	MHO	MUO	ANOVA	Post-hoc Mann-Whitney-Wilcoxongroup comparison (P-values)		
	(mean±SEM)	(mean±SEM)	(mean±SEM)	(P-value)	LH vs.MHO	LH vs.MUO	MHO vs.MUO
*Anthropometric measurements*							
Number of subjects	10(3 men, 7 women)	10(3 men, 7 women)	10(3 men, 7 women)				
Age (yrs)	51±3	50±4	48±2	0.8418			
Weight (kg)	61.9±2.8	86.2±3.4	92.7±6.2	0.0004	0.0001	0.0003	0.5288
Height (cm)	167±3	168±3	167±3	0.9299			
BMI (kg/m^2^)	22.1±0.6	30.6±1.1	33.0±1.9	<0.0001	<0.0001	<0.0001	0.4813
Waist circumference (cm)	77±3	98±3	104±5	0.0002	0.0007	0.0003	0.4359
Hip circumference (cm)	96±1	109±2	113±4	0.0002	0.0003	0.0006	0.5960
Waist-to-hip ratio	0.80±0.02	0.90±0.03	0.92±0.02	0.0075	0.0172	0.0046	0.5787
Fat mass (%)	27.4±2.7	39.8±2.4	39.3±2.4	0.0086	0.0039	0.0115	0.9397
Fat mass (kg)	16.7±1.6	34.1±2.4	36.4±3.4	<0.0001	<0.0001	<0.0001	0.7959
Fat-free mass (%)	72.6±2.7	60.2±2.4	60.8±2.4	0.0086	0.0039	0.0115	0.9397
Fat-free mass (kg)	45.1±3.1	52.0±3.2	56.2±4.4	0.1071			
*Bioclinical measurements*							
Systolic BP (mmHg)	118±4	128±5	128±4	0.1063			
Diastolic BP (mmHg)	75±3	82±2	82±2	0.0664			
Total-c (mmol/L)	4.43±0.30	4.26±0.32	5.34±0.23	0.0169	0.7054	0.0355	0.0073
LDL-c (mmol/L)	2.52±0.25	2.39±0.30	3.27±0.19	0.0401	0.6842	0.0433	0.0232
HDL-c (mmol/L)	1.57±0.08	1.17±0.12	1.04±0.05	0.0018	0.0256	0.0004	0.3634
Total-c/HDL ratio	2.85±0.16	3.81±0.22	5.17±0.18	<0.0001	0.0073	0.0002	0.0010
TG (mmol/L)	0.77±0.05	1.54±0.33	2.26±0.23	0.0004	0.0311	0.0002	0.0524
Fasting glucose (mmol/L)	4.5±0.2	5.0±0.1	5.3±0.2	0.0243	0.0335	0.0171	0.5178
Fasting insulin (pmol/L)	37±17	64±9	118±22	0.0025	0.0129	0.0030	0.0887
HbA1c (%)	5.37±0.07	5.72±0.08	5.73±0.08	0.0051	0.0055	0.0066	0.8775
HOMA-IR	0.65±0.28	1.18±0.16	2.19±0.42	0.0024	0.0143	0.0015	0.0892
HOMA%B	93.7±33.1	105.9±10.1	140.3±17.9	0.0130	0.0337	0.0076	0.2176

Data represented as mean ± SEM. LH, lean healthy; MHO, metabolically healthy obese; MUO, metabolically unhealthy obese; BMI, body mass index; BP, blood pressure; Total-c, total-cholesterol; LDL-c, low-density lipoprotein; HDL-c, high-density lipoprotein; TG, triglycerides; HbA1c, glycosylated haemoglobin; HOMA-IR, homeostatic model assessment for insulin resistance; HOMA%B, homeostatic model assessment for β-cell function. A non-parametric ANOVA Kruskal-Wallis test followed by a post-hoc Mann-Whitney-Wilcoxon test was used to determine significance between groups (P<0.05).

### Inflammatory Marker Analysis

**Figure 1:**
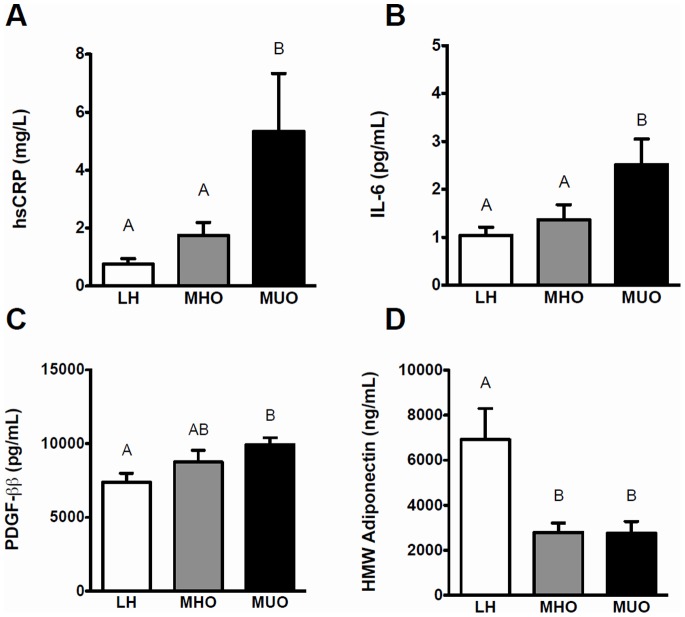
Comparison of mean fasting concentrations of inflammatory markers between groups. (**A**) high sensitivity C-reactive protein (hsCRP, mg/mL), (**B**) interleukin-6 (IL-6, pg/mL), (**C**) platelet-derived growth factor ββ (PDGF-ββ, pg/mL), and (**D**) high molecular weight adiponectin (HMW adiponectin, ng/mL) in lean healthy (LH), metabolically healthy obese (MHO) and metabolically unhealthy obese (MUO) groups (n = 10/group). A non-parametric ANOVA Kruskal-Wallis test followed by a post-hoc Mann-Whitney-Wilcoxon test was used to determine differences between groups. Bars not sharing the same letter are statistically different (P<0.05). White bars = LH; grey bars = MHO; and black bars = MUO.

**Table 2** outlines the concentrations of circulating inflammatory markers measured in the three groups of individuals. The MHO and LH groups had hsCRP and IL-6 levels significantly lower than the MUO group (**Figure 1**), while the MHO group had PDGF-ββ levels intermediate to that of the LH and MUO groups. A similar trend was also seen for IP-10 levels (P = 0.07). In contrast, HMW adiponectin levels were significantly lower in MHO and MUO groups compared to the LH group. No differences were seen in IL-1Ra, IFN-γ, and RANTES levels between the three groups, while TNF-α, MCP-1 and IL-10 levels were not consistently detected due to low circulating levels in several of the study participants.

**Table 2:** Mean circulating concentration of inflammatory markers.

Circulating Marker	LH	MHO	MUO	ANOVA	Post-hoc Mann-Whitney-Wilcoxon group comparison (P-values)		
	(mean±SEM)	(mean±SEM)	(mean±SEM)	(P-value)	LH vs. MHO	LH vs. MUO	MHO vs. MUO
*Pro-Inflammatory markers*							
hsCRP (mg/L)	0.76±0.19	1.75±0.45	5.35±1.99	0.0018	0.1296	0.0009	0.0282
IL-6 (pg/mL)	1.04±0.17	1.37±0.31	2.52±0.53	0.0324	0.7023	0.0169	0.0489
IFN-γ (pg/mL)	402.4±14.3	446.1±17.8	434.6±20.3	0.2611			
IP-10 (pg/mL)	1489±243	1961±311	2363±269	0.0741			
PDGF-ββ (pg/mL)	7373±621	8751±793	9933±468	0.0309	0.1431	0.0089	0.2567
RANTES (pg/mL)	8803±453	8825±433	8292±503	0.9351			
*Anti-Inflammatory markers*							
HMW adiponectin (ng/mL)	6914±1382	2794±412	2752±529	0.0347	0.0288	0.0288	0.5288
IL-1Ra (pg/mL)	675.1±32.0	756.8±40.6	759.1±29.0	0.1978			

Data represented as mean±SEM. LH, lean healthy; MHO, metabolically healthy obese; MUO, metabolically unhealthy obese; hsCRP, high sensitivity C-reactive protein; IL-6, interleukin-6, IFN-γ, interferon γ; IP-10, interferon-γ inducible protein 10; PDGF-ββ, platelet-derived growth factor ββ; RANTES, regulated upon activation normal T-cell expressed and secreted; HMW adiponectin, high molecular weight adiponectin; IL-1Ra, interleukin-1 receptor antagonist. A non-parametric ANOVA Kruskal-Wallis test followed by a post-hoc Mann-Whitney-Wilcoxon test was used to determine significance between groups (P<0.05).

### Fatty Acid Analysis

We first examined total FAs in serum from LH, MHO, and MUO individuals (**Tables 3** **and** **4**). Only FAs that were consistently detected across all participants were included in these analyses. Total FA abundance differed significantly between the three groups (**Table 4**, P = 0.0082). In alignment with our clinical and inflammatory data, total FA abundance in MHO individuals was intermediate to LH and MUO individuals, where LH individuals had the lowest concentration of serum FAs (201.4±5.7 µg/100 µL), followed by MHO (278.5±43.9 µg/100 µL) and MUO (406.0±58.0 µg/100 µL) individuals. Furthermore, 10 individual FAs (myristic acid, myristoleic acid, palmitoleic acid, heptadecenoic acid, stearic acid, oleic acid, linoleic acid, γ-linolenic acid, α-linolenic acid, and arachidonic acid) were found to consistently differ between the three groups when data was expressed in both relative % (**Table 3**) and absolute (**Table 4**) values; indicating high concordance between the two approaches used to report FA levels. Subsequent fractionation of serum lipids revealed that 8 of the 10 aforementioned FAs were also significantly different in the PL and TG fractions (data not shown); indicating good agreement between total and fractionated FA analyses.

**Table 3:** Mean relative percentage values of total fatty acids in serum.

Fatty Acids		LH	MHO	MUO	ANOVA	Post-hoc Mann-Whitney-Wilcoxongroup comparison (P-values)		
		(% ±SEM)	(% ±SEM)	(% ±SEM)	(P-value)	LH vs.MHO	LH vs.MUO	MHO vs.MUO
**14∶0**	***Myristic Acid***	0.625±0.040	0.845±0.081	1.204±0.109	**0.0011**	0.0756	**0.0007**	**0.0257**
15∶0	*Pentadecanoic Acid*	0.230±0.015	0.209±0.010	0.238±0.010	0.1579	0.2730	0.6774	**0.0493**
16∶0	*Palmitic Acid*	20.25±0.39	21.22±0.63	22.25±0.65	0.0539	0.2729	**0.0211**	0.1857
**18∶0**	***Stearic Acid***	7.718±0.183	7.505±0.278	6.730±0.189	**0.0119**	0.5205	**0.0058**	**0.0312**
19∶0	*Nonadecanoic Acid*	0.056±0.006	0.065±0.012	0.054±0.006	0.0799	0.9296	0.2697	0.3268
**20∶0**	***Arachidic Acid***	0.291±0.033	0.206±0.022	0.191±0.013	**0.0437**	0.0890	**0.0172**	0.5706
22∶0	*Behenic Acid*	0.461±0.031	0.399±0.059	0.355±0.044	0.3119	0.5205	0.1041	0.6232
**24∶0**	***Lignoceric Acid***	2.658±0.312	2.373±0.312	1.804±0.100	**0.0344**	0.4274	**0.0091**	0.1405
**14∶1n5**	***Myristoleic Acid***	0.056±0.011	0.086±0.014	0.112±0.016	**0.0092**	**0.0100**	**0.0113**	0.3506
**16∶1n7**	***Palmitoleic Acid***	1.565±0.129	2.160±0.250	2.337±0.147	**0.0137**	0.1212	**0.0022**	0.4274
**17∶1n7**	***Heptadecenoic Acid***	0.146±0.011	0.199±0.018	0.200±0.009	**0.0118**	**0.0257**	**0.0051**	1.0000
**18∶1n9**	***Oleic Acid***	19.56±0.55	21.75±1.25	23.76±0.44	**0.0052**	0.3075	**0.0006**	0.1212
18∶1n7	*Vaccenic Acid*	1.811±0.072	1.888±0.068	1.769±0.048	0.6116	0.7913	0.7337	0.2730
19∶1n9	*cis-Nonadecanoic Acid*	0.282±0.057	0.275±0.040	0.157±0.011	0.0799	0.7913	0.0962	**0.0376**
**22∶1n9**	***Erucic Acid***	0.561±0.062	0.585±0.078	0.369±0.033	**0.0190**	1.0000	**0.0172**	**0.0172**
**24∶1n9**	***Nervonic Acid***	0.072±0.006	0.066±0.010	0.043±0.003	**0.0028**	0.2563	**0.0015**	**0.0190**
**18∶3n3**	***α-Linolenic Acid***	0.705±0.049	0.715±0.056	0.953±0.054	**0.0057**	0.9097	**0.0046**	**0.0091**
20∶5n3	*Eicosapentaenoic Acid (EPA)*	0.955±0.165	0.912±0.098	0.854±0.114	0.7442	0.9698	0.7913	0.3447
22∶3n3	*Docosatrienoic Acid*	0.218±0.017	0.234±0.014	0.208±0.013	0.4339	0.6232	0.4495	0.2263
22∶5n3	*Docosapentaenoic Acid*	0.698±0.036	0.641±0.052	0.591±0.029	0.2104	0.6500	**0.0376**	0.6232
**22∶6n3**	***Docosahexaenoic Acid (DHA)***	0.775±0.042	0.662±0.079	0.526±0.061	**0.0373**	0.4274	**0.0091**	0.1620
**18∶2n6**	***Linoleic Acid***	30.46±0.76	25.75±0.95	26.53±1.00	**0.0030**	**0.0036**	**0.0046**	0.6232
**18∶3n6**	***γ-Linolenic Acid***	0.364±0.049	0.603±0.064	0.553±0.041	**0.0162**	**0.0173**	**0.0140**	0.7054
20∶2n6	*Eicosadienoic Acid*	0.165±0.020	0.131±0.016	0.127±0.012	0.2470	0.1508	0.1735	0.9698
20∶3n6	*Dihomo-γ-Linolenic Acid (DGLA)*	1.476±0.141	1.795±0.095	1.647±0.118	0.2151	0.0890	0.4274	0.3847
**20∶4n6**	***Arachidonic Acid (AA)***	7.383±0.529	8.312±0.662	6.076±0.362	**0.0242**	0.3075	0.0757	**0.0113**
22∶4n6	*Adrenic Acid*	0.229±0.020	0.208±0.027	0.154±0.021	0.0964	0.5708	**0.0376**	0.1620
22∶5n6	*Docosapentaenoic Acid*	0.214±0.017	0.209±0.014	0.183±0.015	0.4921	1.0000	0.2730	0.3843

Total fatty acids (FAs) are reported as relative % values in lean healthy (LH), metabolically healthy obese (MHO), and metabolically unhealthy obese (MUO) groups. A non-parametric ANOVA Kruskal-Wallis test followed by a post-hoc Mann-Whitney-Wilcoxon test was used to determine significance between groups. FAs in bold font were significant in the ANOVA test (P<0.05).

**Table 4:** Mean absolute values of total fatty acids in serum.

Fatty Acids		LH	MHO	MUO	ANOVA	Post-hoc Mann-Whitney-Wilcoxongroup comparison (P-values)		
		(µg/100 µl±SEM)	(µg/100 µl±SEM)	(µg/100 µl±SEM)	(P-value)	LH vs.MHO	LH vs.MUO	MHO vs.MUO
**14∶0**	***Myristic Acid***	1.249±0.070	2.496±0.517	4.962±0.863	**0.0006**	0.1212	**0.0002**	**0.0211**
**15∶0**	***Pentadecanoic Acid***	0.461±0.029	0.577±0.084	1.007±0.180	**0.0141**	0.6224	**0.0040**	0.0537
**16∶0**	***Palmitic Acid***	40.76±1.27	59.78±9.77	92.41±14.79	**0.0060**	0.3075	**0.0010**	0.0890
**18∶0**	***Stearic Acid***	15.57±0.634	20.93±3.603	27.20±3.861	**0.0192**	0.5708	**0.0036**	0.1041
19∶0	*Nonadecanoic Acid*	0.114±0.014	0.210±0.064	0.240±0.055	0.4702	0.8932	0.2496	0.4122
20∶0	*Arachidic Acid*	0.586±0.070	0.535±0.077	0.753±0.114	0.1032	0.3445	0.1400	0.0640
**22∶0**	***Behenic Acid***	0.926±0.067	0.941±0.082	1.231±0.074	**0.0176**	0.7054	**0.0113**	**0.0257**
24∶0	*Lignoceric Acid*	5.405±0.724	5.912±0.605	7.462±1.356	0.3770	0.3447	0.2123	0.6232
**14∶1n5**	***Myristoleic Acid***	0.108±0.020	0.285±0.078	0.475±0.090	**0.0037**	**0.0098**	**0.0038**	0.2468
**16∶1n7**	***Palmitoleic Acid***	3.132±0.240	6.819±1.820	9.833±1.699	**0.0026**	0.1620	**0.0003**	0.1212
**17∶1n7**	***Heptadecenoic Acid***	0.294±0.026	0.566±0.098	0.816±0.123	**0.0016**	0.0632	**0.0004**	0.1040
**18∶1n9**	***Oleic Acid***	39.28±1.15	61.54±10.05	97.14±14.41	**0.0031**	0.3075	**0.0003**	0.0890
**18∶1n7**	***Vaccenic Acid***	3.638±0.159	5.388±0.944	7.172±1.062	**0.0126**	0.4055	**0.0017**	0.1405
19∶1n9	*cis-Nonadecanoic Acid*	0.571±0.117	0.736±0.138	0.653±0.108	0.5545	0.2413	0.6499	0.7909
22∶1n9	*Erucic Acid*	1.130±0.131	1.555±0.266	1.435±0.203	0.5825	0.4727	0.3642	0.7337
24∶1n9	*Nervonic Acid*	0.145±0.014	0.169±0.022	0.177±0.030	0.8143	0.5426	0.6763	0.9696
**18∶3n3**	***α-Linolenic Acid***	1.414±0.100	2.097±0.438	3.937±0.622	**0.0061**	0.5708	**0.0017**	**0.0342**
20∶5n3	*Eicosapentaenoic Acid (EPA)*	1.960±0.374	2.473±0.455	3.725±0.948	0.1917	0.3075	0.0962	0.3642
**22∶3n3**	***Docosatrienoic Acid***	0.441±0.039	0.647±0.097	0.795±0.083	**0.0138**	0.0962	**0.0041**	0.2730
22∶5n3	*Docosapentaenoic Acid*	1.410±0.090	1.778±0.309	2.400±0.365	0.0871	0.6775	**0.0283**	0.1618
22∶6n3	*Docosahexaenoic Acid (DHA)*	1.556±0.083	1.628±0.151	1.851±0.130	0.2076	0.8498	0.0818	0.2404
**18∶2n6**	***Linoleic Acid***	61.45±2.47	71.81±12.45	105.8±14.37	**0.0165**	0.5708	**0.0058**	**0.0376**
**18∶3n6**	***γ-Linolenic Acid***	0.728±0.100	1.675±0.326	2.204±0.333	**0.0006**	**0.0046**	**0.0006**	0.2123
20∶2n6	*Eicosadienoic Acid*	0.338±0.047	0.391±0.089	0.502±0.069	0.2130	0.7052	0.1403	0.1508
**20∶3n6**	***Dihomo-γ-Linolenic Acid (DGLA)***	2.937±0.249	5.176±1.076	6.237±0.614	**0.0008**	**0.0257**	**0.0003**	0.0962
**20∶4n6**	***Arachidonic Acid (AA)***	14.97±1.334	21.40±2.303	24.33±3.513	**0.0430**	**0.0257**	**0.0452**	1.0000
22∶4n6	*Adrenic Acid*	0.457±0.032	0.510±0.053	0.522±0.038	0.5209	0.4494	0.2894	0.7911
**22∶5n6**	***Docosapentaenoic Acid***	0.431±0.037	0.582±0.091	0.700±0.078	**0.0408**	0.2897	**0.0101**	0.2411
**Total**		201.4±5.7	278.5±43.9	406.0±58.0	**0.0082**	0.2730	**0.0022**	0.0757

Total FAs are reported as absolute (µg/100 µL of serum) values in lean healthy (LH), metabolically healthy obese (MHO), and metabolically unhealthy obese (MUO) groups. A non-parametric ANOVA Kruskal-Wallis test followed by a post-hoc Mann-Whitney-Wilcoxon test was used to determine significance between groups. FAs in bold font were significant in the ANOVA test (P<0.05).

**Figure 2:**
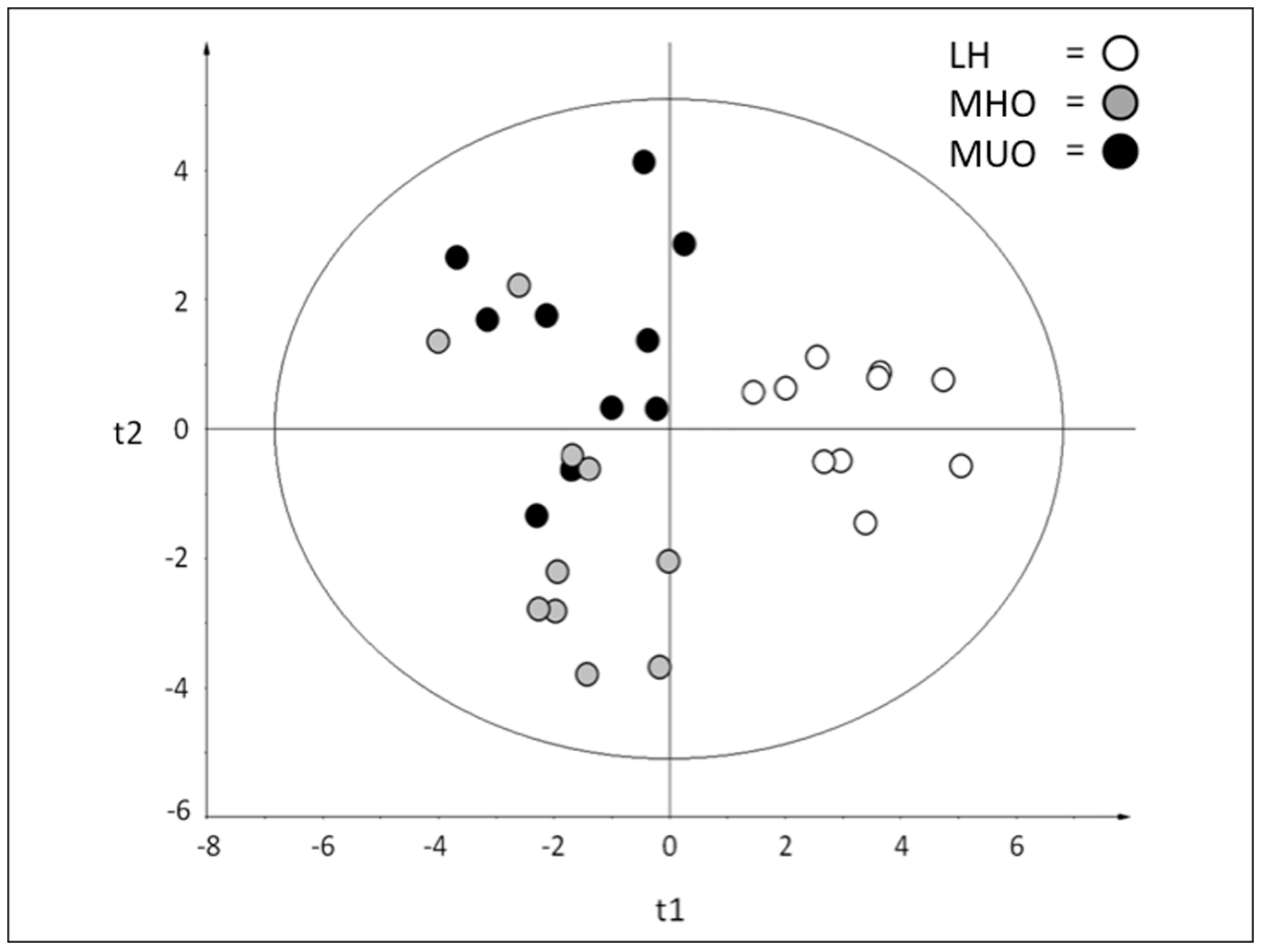
Orthogonal Projections to Latent Structures-Discriminatory Analysis (OPLS-DA) corresponding to the fatty acid profile from serum phospholipid and triglyceride fractions. The fatty acid (FA) profiles from serum phospholipid (PL) and triglyceride (TG) fractions (expressed as relative % values) were analyzed for their ability to distinguish lean healthy (LH), metabolically healthy obese (MHO), and metabolically unhealthy obese (MUO) individuals (n = 10/group). The OPLS-DA parameters obtained revealed 58% of inter-group variability and 32% of prediction ability (R^2^Y_cum_ = 0.58, Q^2^Y_cum_ = 0.32, CV-ANOVA = 0.05).

We next examined whether total FA profiles (expressed as either relative % or absolute values), as well as FA profiles from PL and TG fractions (expressed as either relative % or absolute values), could be used to distinguish the three groups. OPLS-DA modelling indicated that the FA profile from PL and TG fractions expressed as relative % values provided the best combination of fit (R^2^Y_cum_ = 0.58) and predictive ability (Q^2^Y_cum_ = 0.32, CV-ANOVA = 0.05) to distinguish the three groups (**Table S1**). As seen in **Figure 2**, OPLS-DA was able to clearly discriminate LH from the MHO and MUO groups, while the MHO and MUO groups showed a small degree of overlap. Subsequent analysis identified a panel of nine FAs from the PL and TG fractions (i.e., VIP>1) that could discriminate the three groups (**Table S2**). The nine FAs included: PL-linoleic acid, PL-dihomo-γ-linolenic acid (DGLA), PL-arachidonic acid, PL-erucic acid, TG-myristic acid, TG-palmitic acid, TG-stearic acid, TG-oleic acid, and TG-erucic acid. These nine FA may have potential to serve, collectively, as a biomarker to distinguish MHO from MUO groups.

**Figure 3:**
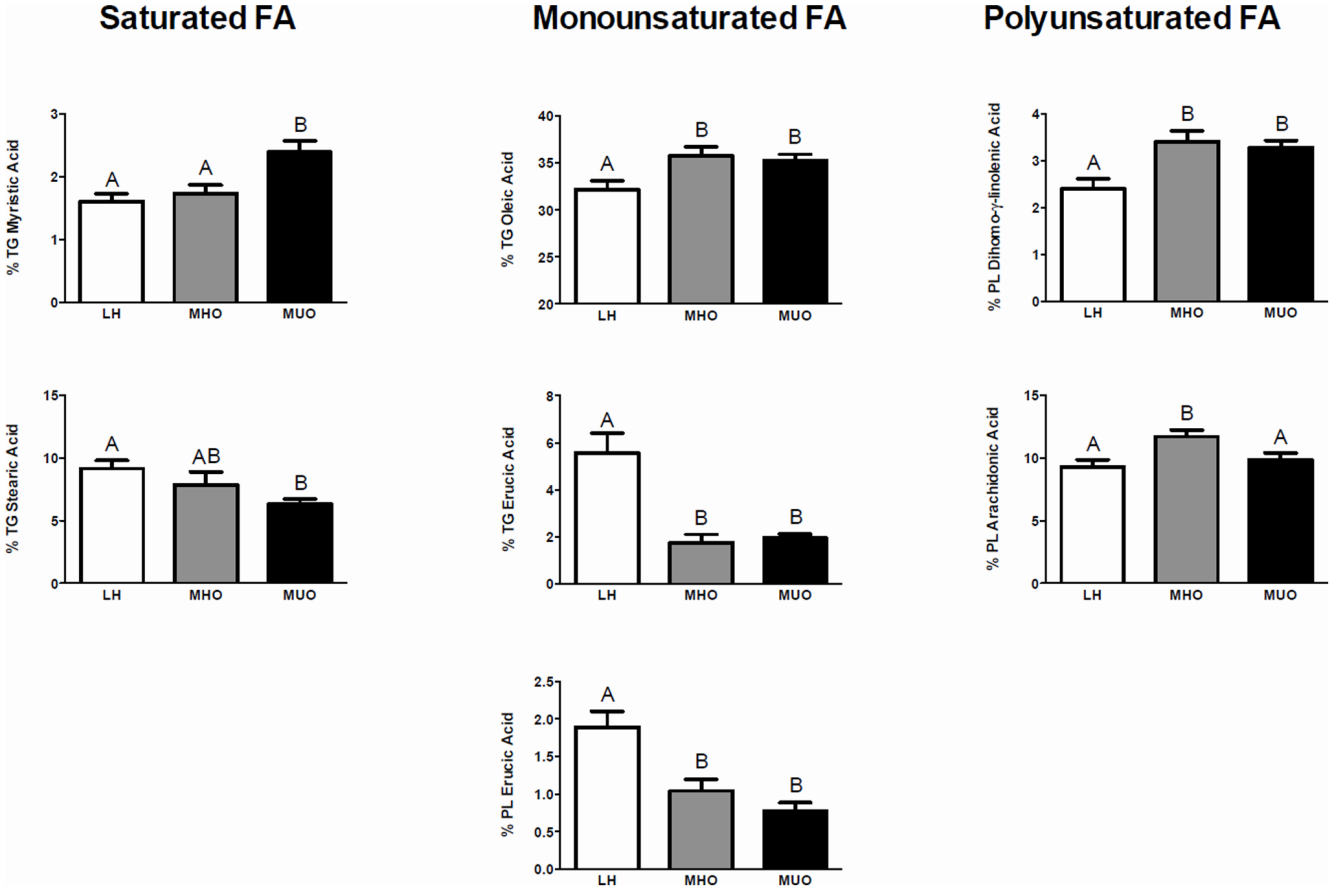
Mean relative percentage values of fatty acids identified in the serum phospholipid and triglyceride fraction. Fatty acids (FAs) meeting a VIP>1 in our OPLS-DA model were individually assessed between the three groups using a non-parametric ANOVA Kruskal-Wallis test followed by a post-hoc Mann-Whitney-Wilcoxon test. Bars not sharing the same letter are statistically different (P<0.05). White bars = LH; grey bars = MHO; and black bars = MUO.

Examining these nine FAs individually revealed that seven of them differed significantly between the three groups (**Figure 3**). The MHO and LH groups had significantly lower levels of serum TG-myristic acid compared to the MUO group. MHO and MUO groups had higher levels of TG-oleic acid and PL-DGLA and lower levels of TG- and PL-erucic acid compared to the LH group. MHO individuals had a level of TG-stearic acid that was intermediate to that of the LH and MUO groups. Finally, the MHO group had significantly greater levels of PL-arachidonic acid compared to both the LH and MUO groups. No changes were detected between the three groups for PL-linoleic acid and TG-palmitic acid.

Finally, we used linear regression to examine the relationships between the aforementioned seven FAs and measures of adiposity (BMI and body fat %). PL-DGLA was positively associated with both BMI (r^2^ = 0.252, p = 0.006) and body fat % (r^2^ = 0.231, p = 0.008), while TG-oleic acid was only positively associated with BMI (r^2^ = 0.194, p = 0.030). In contrast, TG- and PL-erucic acid were inversely associated with both BMI (r^2^ = 0.326, p = 0.001 and r^2^ = 0.531, p<0.001; respectively) and body fat % (r^2^ = 0.249, p = 0.006 and r^2^ = 0.350, p = 0.001; respectively), while TG-stearic acid was only inversely associated with BMI (r^2^ = 0.191, p = 0.018). No significant associations were observed between markers of adiposity and PL-arachidonic acid or TG-myristic acid.

## Discussion

The current study makes an important contribution to the growing field of research aimed at better understanding the clinical and molecular basis underlying the MHO phenotype. We have demonstrated that MHO individuals have an inflammatory state comparable to that of LH individuals. Furthermore, underlying this reduced inflammation is a distinct FA profile comprised of saturated, monounsaturated and n-6 polyunsaturated fats. Together, our results demonstrate that MHO individuals are metabolically healthier than their MUO counterparts and that the reduced inflammatory state may stem from a more favourable FA profile.

There is currently no consensus for classifying individuals as MHO [23]. We used the classification criteria initially proposed by Karelis *et al.* [20] with some minor modifications to account for known sex differences. First, we used newly proposed sex-specific cut-off values for BMI, where obesity is defined as ≥28 kg/m^2^ in men and ≥24 kg/m^2^ in women, rather than a general cut-off of 30 kg/m^2^ [19]. These new cut-offs were found to correlate better with body fat % and thus provide a more accurate assessment of adiposity status. Second, we accounted for known sex-differences in HDL-c, where >1.0 mmol/L for males and >1.3 mmol/L for females were used to assess metabolic health. Finally, we ensured that study participants were not taking medications to normalize hypertriglyceridemia or type 2 diabetes, as this would have created a significant confounder. Using these classification criteria we were able to successfully distinguish LH, MHO, and MUO groups, as seen by group differences in anthropometric and bioclinical measurements. Notably, the MHO group had levels of Total-c and LDL-c comparable to the LH group. Further, we found a trend for reduced measures of fasted insulin and HOMA-IR in MHO versus MUO individuals, suggesting that the MHO group was more insulin sensitive compared to their MUO counterparts. This agrees with past studies in which glucose clamps were used to assess insulin sensitivity in these groups [14]. Collectively, these results suggest that our classification criteria successfully identified MHO individuals from within the DRA cohort.

A number of circulating anti- and pro-inflammatory markers were measured in our three groups in order to determine differences in inflammatory status. We found that circulating levels of hsCRP and IL-6 were significantly reduced in the MHO and LH groups compared to the MUO group; thus, agreeing with previous reports in the area [5], [7], [8]. Further, MHO subjects had intermediate levels of PDGF-ββ compared to the LH and MUO groups. The same trend was observed for the pro-inflammatory marker IP-10, where MHO individuals had an intermediate level compared to the LH and MUO groups. Together, our results demonstrate that MHO individuals have a reduced inflammatory status compared to their MUO counterparts. The discovery of PDGF-ββ is intriguing due to its suspected contribution to the development of atherogenesis [24]. This aligns with the fact that MHO and MUO subjects in our cohort were strongly distinguished by their lipid profiles (i.e., Total-c and LDL-c), which are routinely used to assess an individual’s risk for atherogenesis [25]. It is therefore not surprising that PDGF-ββ levels could distinguish the MHO from MUO group. Further, our findings suggest that subsequent investigations of the MHO phenotype should assess hepatic function. Indeed, hsCRP is primarily produced by the liver and regulated by IL-6 [2], while this organ also plays a central role in lipoprotein production [26]. Thus, our results corroborate recent findings suggesting that studying the liver may provide further clues to help unravel the MHO phenotype [27].

Given that the MHO group was found to be more insulin sensitive (as suggested by fasted insulin and HOMA-IR values) compared to the MUO group, it was somewhat surprising to find that HMW adiponectin levels were comparable between these two groups. Adiponectin is a protein produced by adipose tissue and has known insulin-sensitizing [28] and anti-inflammatory [2] properties. Our adiponectin results are similar to those reported by Telle-Hansen *et al.*, who had an equivalently sized cohort and used similar classification criteria to identify MHO individuals [15]. However, our findings do not agree with those of other studies showing that MHO individuals had higher adiponectin levels compared to MUO individuals [29]–[33]. This suggests that the role of adiponectin in MHO individuals remains unclear.

Considerable evidence has demonstrated a strong relationship between FAs and inflammation [9], [34]. Specifically, saturated [35] and *trans* [36] fats tend to be positively associated with inflammation, while monounsaturated [37] and polyunsaturated [38] fats tend to be inversely associated; however, recent evidence suggests this is overly simplified. For example, we and others have reported distinct relationships between saturated fats and inflammation, where myristic acid and palmitic acid were positively associated with inflammation, while stearic acid was inversely associated with inflammation [39], [40]. In light of these recent findings we aimed to examine whether the reduced inflammatory state seen in MHO individuals compared to MUO individuals could be related to underlying differences in FA profiles. This was accomplished in two steps: (1) we analyzed total serum FA profiles, as well as FA profiles in PL and TG fractions, by discriminant analysis modelling to determine whether we could identify a panel of FAs that could differentiate the three groups; and (2) FAs that had significant contributions (i.e., VIP>1) in the discriminant analysis were examined individually to determine group differences.

In alignment with our clinical and inflammatory data, we found that MHO individuals had an intermediate level of total FAs compared to LH and MUO individuals. This reinforces the relevance of studying FA profiles in MHO individuals, as this may well contribute to their healthier metabolic phenotype. Discriminant analysis modelling was conducted to determine if serum FA profiles (either total or fractionated) could distinguish LH, MHO, and MUO individuals. The OPLS-DA scatter plot (**Figure 2**) revealed that the FA profile from PL and TG fractions (expressed as relative %) could distinguish the three groups better than total serum FAs. Specifically, FAs in the PL and TG fractions could explain 58% of the variability (R^2^Y_cum_) between the three groups, while the predictability of the model (Q^2^Y_cum_) was found to be 32%. This aligns with previous work by Fernández-Real *et al*., which reported that FAs expressed as relative % values distinguished lean and obese individuals more strongly than when FAs were expressed as absolute values [12]. The outcome of our discriminant analysis modelling was also similar to that previously shown by Donovan *et al.,* who reported a similar scatter plot distribution when using FA profiling to distinguish lean from morbidly obese individuals [41]. Of interest, it was noted that while the LH group in our study was clearly distinct from both of the obese groups, the classification of MHO and MUO individuals showed some overlap. This suggests that despite having distinct FA profiles, some specific FA characteristics are shared between the MHO and MUO groups. For example, Petersson and colleagues demonstrated that a number of FAs (such as oleic acid and DGLA) associated more strongly with adiposity than inflammation [42], [43]. This aligns with the results from our regression analyses, which showed that PL-DGLA, PL-erucic acid, TG-stearic acid, TG-oleic acid, and TG-erucic acid were all associated with measures of adiposity (BMI and body fat %). Given that our MHO and MUO groups are matched for various measures of adiposity, it is therefore not surprising to see a degree of overlap in their FA profiles. Nevertheless, our modelling approach led to the identification of a panel of nine FAs that was able to differentiate the three groups. This panel consisted of TG-myristic acid, TG-palmitic acid, TG-stearic acid, TG-oleic acid, TG-erucic acid, PL-linoleic acid, PL-DGLA, PL-arachidonic acid (AA), and PL-erucic acid. It is important to note that subjects were fasted for 12 hours prior to sample collection, thereby minimizing the possibility that our results reflect acute differences in dietary habits. Since the panel of nine FAs was able to distinguish the three groups, we next examined these FAs individually in order to determine if a pattern existed. Intriguingly, our data revealed that MHO individuals have a more favourable saturated FA profile compared to MUO individuals. It does not appear that monounsaturated fats play a key role in distinguishing MHO from MUO, while twenty-carbon n-6 polyunsaturated fats had a tendency to be higher in MHO compared to MUO. Together, our findings suggest that FAs may indeed be associated with the distinct inflammatory status of the MHO and MUO groups.

Concerning saturated FAs (SFA), the MHO and LH groups had similar levels of TG-myristic acid, which were significantly lower than those observed in the MUO group. This is intriguing in light of previous work by Fernández-Real *et al.* who reported a positive association between fasting serum myristic acid and IL-6 in a group of 232 adults [12]. Further, a reduction in the level of plasma TG enriched with myristic acid was previously shown to be strongly correlated with improvements in insulin sensitivity in obese subjects during a weight-loss intervention [44]. Interestingly, we found no evidence of an association between TG-myristic acid and measures of adiposity status; suggesting that lower levels of TG-myristic acid may have a causal role in both the reduced inflammatory status and improved insulin sensitivity observed in MHO individuals. Conversely, the levels of TG-stearic acid in the LH and MHO were elevated compared to the MUO group. This agrees with previous work from our lab in which we reported an inverse relationship between circulating stearic acid levels and markers of inflammation in young lean female adults from the Toronto Nutrigenomics and Health Study cohort [11]. Taken together, the lower levels of myristic acid and the elevated levels of stearic acid in MHO individuals (compared to MUO individuals) coincide with a reduced inflammatory state and suggest that the distinct pattern of SFA seen in MHO versus MUO individuals may provide an explanation for the inflammatory characteristics of these two groups.

In MHO individuals, circulating levels of monounsaturated FAs (MUFA) TG-oleic, TG-erucic, and PL-erucic acids were similar to that observed in MUO individuals, but different from LH individuals. Oleic acid was previously shown to be elevated in obese individuals and those at increased risk of metabolic syndrome [45], [46]. Moreover, detecting increased oleic acid in the TG fraction (as opposed to the PL fraction) of obese individuals agrees with previous work by Gil-Campos *et al*. [46]. In comparison, the similar levels of erucic acid detected in MHO and MUO individuals were surprising in light of a recent study showing that elevated plasma levels of PL-erucic acid were associated with an increased risk of heart failure [47]. However, investigations of erucic acid in humans are limited [48] and it is, therefore, difficult to explain our data showing significant reductions in erucic acid (in both the TG and PL fractions) in MHO and MUO individuals compared to LH individuals. Considering the improved lipid status of MHO individuals compared to MUO individuals, we would have expected a difference in erucic acid levels that would reflect the varying risk for cardiovascular complications. This suggests that further studies examining erucic acid are required, as current evidence is scarce and we presently possess only a limited understanding of its role in metabolism and obesity.

The levels of DGLA and AA, two n-6 polyunsaturated FAs (PUFA), were found to differ in the PL fraction between the three groups, with LH individuals having the lowest levels of both. The higher level of PL-DGLA seen in both obese groups agrees with recent work showing a positive association between BMI and DGLA [49]. Interestingly, we also found that MHO individuals had significantly higher levels of PL-AA compared to both LH and MUO individuals. While the elevated level of PL-AA in the MHO group coincides with past work demonstrating a positive association between AA and BMI, the equivalent levels of AA in the LH and MUO were surprising and warrant further investigation [49]. Moreover, the reason for the distinct pattern of these two n-6 PUFA is unclear; however, it is tempting to speculate that this may alter the balance of pro- and anti-inflammatory eicosanoids. Indeed, while DGLA is a precursor for anti-inflammatory series-1 eicosanoids, AA is a precursor for pro-inflammatory series-2 eicosanoids [50]. Future work will help clarify how this distinct pattern of n-6 PUFA influences eicosanoid biosynthesis, and whether this is an additional mechanism by which inflammation is reduced in MHO individuals.

Our understanding of the metabolic basis for MHO remains in its infancy. However, the current study has shed light on this complex area by showing that this distinct subgroup of obese individuals has a more favourable FA profile compared to their MUO counterparts. While the current study has focused on the two dominant lipid fractions in serum (i.e., PL and TG), the interesting results presented here lend strong support to conduct a more comprehensive lipidomic analysis to examine other lipid fractions (e.g., non-esterified fatty acids, cholesterol esters, eicosanoids, etc.) that may provide additional clues to help unravel the MHO phenotype. Due to the known links between FAs and inflammation, it is intriguing to postulate that tailoring recommendations regarding dietary fat intake (e.g., increasing the consumption of foods rich in stearic acid) may help to prevent the downstream metabolic consequences associated with obesity-related inflammation. This work reinforces that continued efforts in this area are necessary in order to elucidate how the distinct FA profile of MHO individuals contributes to their reduced inflammatory status.

## Supporting Information

Table S1 **Orthogonal Projections to Latent Structures-Discriminant Analysis (OPLS-DA) of Fatty Acid Profiles.** To distinguish the three groups, OPLS-DA analyses were conducted using different fatty acid (FA) datasets corresponding to either total serum FA profiles (expressed as either relative % or absolute values), or FA profiles from phospholipid and triglyceride fractions (expressed as either relative % or absolute values). Values for R^2^X_cum_ and R^2^Y_cum_ indicate the variation in the X (i.e., FAs) and Y (i.e., the three groups: LH, MHO, and MUO) parameters that are explained by the model. Q^2^Y_cum_ represents the model’s ability to reliably predict the Y parameter. CV-ANOVA = Analysis of Variance of Cross Validated residuals.
(DOC)

Table S2 **Mean relative percentage of phospholipid and triglyceride fatty acids in serum.** Relative fatty acid (FA) values were calculated as a % of all FAs detected and are reported as relative % FA ± SEM. The FAs in bold font have a Variable of Importance in Projection (VIP) greater than 1 (as determined with an OPLS-DA), which indicates that they are of importance when distinguishing the lean healthy (LH), metabolically healthy obese (MHO) and metabolically unhealthy obese (MUO) groups.
(DOC)
