# Supplementary material for: A Distinct Fatty Acid Profile Underlies the Reduced Inflammatory State of Metabolically Healthy Obese Individuals
Source: PLoS One. 2014 Feb 10;9(2):e88539. doi: 10.1371/journal.pone.0088539 (PMC3919777; doi:10.1371/journal.pone.0088539)
Supplement: Table S1 — Orthogonal Projections to Latent Structures-Discriminant Analysis (OPLS-DA) of Fatty Acid Profiles. To distinguish the three groups, OPLS-DA analyses were conducted using different fatty acid (FA) datasets corresponding to either total serum FA profiles (expressed as either relative % or absolute values), or FA profiles from phospholipid and triglyceride fractions (expressed as either relative % or absolute values). Values for R2Xcum and R2Ycum indicate the variation in the X (i.e., FAs) and Y (i.e., the three groups: LH, MHO, and MUO) parameters that are explained by the model. Q2Ycum represents the model’s ability to reliably predict the Y parameter. CV-ANOVA = Analysis of Variance of Cross Validated residuals. (DOC) [file pone.0088539.s001.doc]

**Table S1**: **Orthogonal Projections to Latent Structures-Discriminant Analysis (OPLS-DA) of Fatty Acid Profiles**.

| **Dataset used for OPLS-DA** | **OPLS-DA Parameter** | | | |
| --- | --- | --- | --- | --- |
|  | R^2^X_cum_ | R^2^Y_cum_ | Q^2^Y_cum_ | CV-ANOVA |
| *Total Fatty Acids* | | | | |
| Absolute (μg / 100 μL of serum) | 0.849 | 0.158 | 0.103 | 0.54 |
| Relative (%) | 0.743 | 0.463 | 0.252 | 0.09 |
|  | | | | |
| *Fatty Acids from Phospholipid and Triglyceride Fractions Only* | | | | |
| Absolute (μg / 100 μL of serum) | 0.666 | 0.608 | 0.235 | 0.40 |
| Relative (%) | 0.506 | 0.579 | 0.321 | 0.05 |
