# Supplementary material for: A Distinct Fatty Acid Profile Underlies the Reduced Inflammatory State of Metabolically Healthy Obese Individuals
Source: PLoS One. 2014 Feb 10;9(2):e88539. doi: 10.1371/journal.pone.0088539 (PMC3919777; doi:10.1371/journal.pone.0088539)
Supplement: Table S2 — Mean relative percentage of phospholipid and triglyceride fatty acids in serum. Relative fatty acid (FA) values were calculated as a % of all FAs detected and are reported as relative % FA ± SEM. The FAs in bold font have a Variable of Importance in Projection (VIP) greater than 1 (as determined with an OPLS-DA), which indicates that they are of importance when distinguishing the lean healthy (LH), metabolically healthy obese (MHO) and metabolically unhealthy obese (MUO) groups. (DOC) [file pone.0088539.s002.doc]

**Table S2**: **Mean relative percentage of phospholipid and triglyceride fatty acids in serum**.

| **Fatty Acids** | | **LH** | **MHO** | **MUO** | **VIP** |
| --- | --- | --- | --- | --- | --- |
|  | | (% ± SEM) | (% ± SEM) | (% ± SEM) |  |
| *Phospholipids (PL)* | | | | | |
| 14:0 | *Myristic Acid* | 0.35 ± 0.02 | 0.41 ± 0.02 | 0.47 ± 0.03 | 0.39 |
| 15:0 | *Pentadecanoic Acid* | 0.25 ± 0.02 | 0.23 ± 0.01 | 0.23 ± 0.01 | 0.10 |
| 16:0 | *Palmitic Acid* | 28.26 ± 0.45 | 28.76 ± 0.44 | 28.97 ± 0.27 | 0.54 |
| 18:0 | *Stearic Acid* | 15.66 ± 0.44 | 16.39 ± 0.44 | 16.52 ± 0.29 | 0.80 |
| 20:0 | *Arachidic Acid* | 0.46 ± 0.03 | 0.39 ± 0.02 | 0.35 ± 0.01 | 0.25 |
| 22:0 | *Behenic Acid* | 1.37 ± 0.06 | 1.05 ± 0.05 | 1.00 ± 0.06 | 0.58 |
| 24:0 | *Lignoceric Acid* | 1.03 ± 0.07 | 0.82 ± 0.08 | 0.74 ± 0.06 | 0.42 |
| 16:1n7 | *Palmitoleic Acid* | 0.50 ± 0.04 | 0.42 ± 0.08 | 0.59 ± 0.06 | 0.47 |
| 17:1n7 | *Heptadecenoic Acid* | 0.11 ± 0.03 | 0.10 ± 0.01 | 0.07 ± 0.00 | 0.14 |
| 18:1n9 | *Oleic Acid* | 8.94 ± 0.39 | 8.6 ± 0.33 | 8.94 ± 0.31 | 0.33 |
| 18:1n7 | *Vaccenic Acid* | 1.73 ± 0.11 | 1.85 ± 0.08 | 1.68 ± 0.08 | 0.46 |
| **22:1n9** | ***Erucic Acid*** | **1.89 ± 0.21** | **1.04 ± 0.15** | **0.83 ± 0.10** | **1.28** |
| 24:1n9 | *Nervonic Acid* | 1.50 ± 0.19 | 1.5 ± 0.12 | 1.22 ± 0.11 | 0.27 |
| 18:3n3 | *α-Linolenic Acid* | 0.72 ± 0.09 | 0.49 ± 0.04 | 0.63 ± 0.08 | 0.43 |
| 20:5n3 | *Eicosapentaenoic Acid (EPA)* | 0.88 ± 0.16 | 0.93 ± 0.07 | 1.21 ± 0.20 | 0.58 |
| 22:3n3 | *Docosatrienoic Acid* | 0.33 ± 0.02 | 0.35 ± 0.02 | 0.33 ± 0.02 | 0.13 |
| 22:5n3 | *Docosapentaenoic Acid* | 0.95 ± 0.05 | 0.91 ± 0.09 | 0.96 ± 0.05 | 0.15 |
| 22:6n3 | *Docosahexaenoic Acid (DHA)* | 3.74 ± 0.60 | 3.26 ± 0.54 | 3.08 ± 0.35 | 0.51 |
| **18:2n6** | ***Linoleic Acid*** | **18.91 ± 0.46** | **16.72 ± 0.71** | **18.44 ± 0.76** | **1.66** |
| **20:3n6** | ***Dihomo-γ-Linolenic acid (DGLA)*** | **2.41 ± 0.21** | **3.42 ± 0.22** | **3.37 ± 0.20** | **1.12** |
| **20:4n6** | ***Arachidonic Acid (AA)*** | **9.28 ± 0.58** | **11.71 ± 0.52** | **9.77 ± 0.54** | **1.97** |
| 22:4n6 | *Adrenic Acid* | 0.51 ± 0.03 | 0.46 ± 0.02 | 0.38 ± 0.03 | 0.27 |
| 22:5n6 | *Docosapentaenoic Acid* | 0.23 ± 0.02 | 0.22 ± 0.02 | 0.23 ± 0.02 | 0.09 |
| *Triglycerides (TG)* | |  |  |  |  |
| **14:0** | ***Myristic Acid*** | **1.61 ± 0.12** | **1.74 ± 0.14** | **2.39 ± 0.18** | **1.30** |
| **16:0** | ***Palmitic Acid*** | **27.06 ± 0.64** | **29.21 ± 0.67** | **29.70 ± 1.00** | **1.72** |
| **18:0** | ***Stearic Acid*** | **9.15 ± 0.64** | **7.86 ± 1.04** | **6.32 ± 0.40** | **1.78** |
| 16:1n7 | *Palmitoleic Acid* | 3.18 ± 0.23 | 3.13 ± 0.49 | 3.20 ± 0.28 | 0.09 |
| **18:1n9** | ***Oleic Acid*** | **32.16 ± 0.96** | **35.76 ± 0.99** | **35.24 ± 0.69** | **2.11** |
| 18:1n7 | *Vaccenic Acid* | 2.23 ± 0.09 | 2.90 ± 0.12 | 2.68 ± 0.13 | 0.96 |
| **22:1n9** | ***Erucic Acid*** | **5.66 ± 0.88** | **1.74 ± 0.36** | **1.94 ± 0.17** | **2.42** |
| 18:3n3 | *α-Linolenic Acid* | 2.14 ± 0.36 | 1.47 ± 0.12 | 1.99 ± 0.13 | 0.87 |
| 18:2n6 | *Linoleic Acid* | 14.56 ± 0.94 | 14.5 ± 0.59 | 14.88 ± 0.64 | 0.43 |
| 20:4n6 | *Arachidonic Acid (AA)* | 1.78 ± 0.25 | 1.39 ± 0.17 | 1.28 ± 0.20 | 0.56 |
